# A critical appraisal of clinical epigenetics

**DOI:** 10.1186/s13148-022-01315-6

**Published:** 2022-07-28

**Authors:** Bernhard Horsthemke

**Affiliations:** Institut für Humangenetik, Universität Duisburg-Essen, Hufelandstrasse 55, 45122 Essen, Germany

**Keywords:** Epigenetics, Methylation, Chromatin modifications, Epimutations, Transgenerational epigenetics, Genetic variation, Genomic errors, Cell mixture

## Abstract

Modern epigenetics emerged about 40 years ago. Since then, the field has rapidly grown. Unfortunately, this development has been accompanied by certain misconceptions and methodological shortcomings. A profound misconception is that chromatin modifications are a distinct layer of gene regulation that is directly responsive to the environment and potentially heritable between generations. This view ignores the fact that environmental factors affect gene expression mainly through signaling cascades and the activation or repression of transcription factors, which recruit chromatin regulators. The epigenome is mainly shaped by the DNA sequence and by transcription. Methodological shortcomings include the insufficient consideration of genetic variation and cell mixture distribution. Mis- and overinterpretation of epigenetic data foster genetic denialism ("We can control our genes") and epigenetic determinism ("You are what your parents ate"). These erroneous beliefs can be overcome by using precise definitions, by raising the awareness about methodological pitfalls and by returning to the basic facts in molecular and cellular biology.

## Introduction

Modern epigenetics emerged about 40 years ago. Since then, the field has rapidly grown and significantly contributed to the understanding of human disease, also thanks to *Clinical Epigenetics*. As a witness of this development (I wrote my first epigenetic paper in 1989 [1] and since then have contributed more than 100 papers to the field), I am concerned about certain misconceptions and methodological shortcomings in epigenetic research.

*Clinical Epigenetics* was inaugurated in 2010. Until 12 February 2022, 1,334 articles have been published in the journal [2]. A search on the journal's homepage for "methylation", "histone" and "miRNA" retrieved 1246, 590 and 210 results, respectively [3]. Thus, the majority of articles deal with chromatin modifications (DNA methylation and histone modifications), which are at the heart of modern epigenetics. This is in line with the definition by A. Bird, who defined epigenetics as "the structural adaptation of chromosomal regions so as to register, signal or perpetuate altered activity states" [4]. Good examples are the silencing of retrogenes, the inactivation of the X-chromosome in female mammals and genomic imprinting.

I do not like the most common definition of epigenetics, which defines epigenetics as the study of mitotically and/or meiotically heritable changes in gene function that are not caused by a change in DNA sequence [5]. This is a negative and imprecise definition. It blurs the distinction between cellular memory, a concept first proposed by Nanney in 1958 [6], and heredity, i.e. the transmission of genetic information from one generation to the next. Furthermore, some researchers include miRNAs and other RNAs as well as RNA modifications, but RNA is a diffusible molecule and carries DNA-based sequence information, which is a completely different story, also with regard to transgenerational inheritance (a search for "transgenerational" retrieved 61 results in *Clinical Epigenetics*). The oocyte is full of RNAs and even sperm carry small RNAs; the contribution of these RNAs to the zygote and early cleavage states should not be confused with epigenetic inheritance; at best this RNA-based inheritance. I hail the Editors' decision that "manuscripts focusing on differential RNA expression levels (coding or non-coding) or on RNA modifications cannot be considered for publication in *Clinical Epigenetics* since these aspects are not part of epigenetics per se" [3].

In the following, I will discuss some misconceptions and methodological shortcomings in epigenetic research as well as the role of epigenetics in disease.

## Misconceptions

A search for "environment", "stress" and "diet" retrieved 433, 381 and 221 results in *Clinical Epigenetics*, respectively. Many of these articles as well as related articles in other journals explicitly or implicitly assume that chromatin modifications are a distinct layer of gene regulation that is directly responsive to the environment and potentially heritable between generations. Here is an example from a paper in *Clinical Epigenetics*: "Epigenetics is a mechanism that regulates gene expression independently of the underlying DNA sequence, relying instead on the chemical modification of DNA and histone proteins. … Epigenetics is a reversible system that can be affected by various environmental factors, such as drugs, nutrition, and mental stress … In this review, we discuss the nature of epigenetic disorders … on the basis of recent findings: (1) susceptibility of the conditions to environmental factors, (2) treatment by taking advantage of their reversible nature, and (3) transgenerational inheritance of epigenetic changes, that is, acquired adaptive epigenetic changes that are passed on to offspring." [7]

What the authors call "a new concept of clinical genetics" [7] is not compatible with basic facts in molecular and cellular biology (see below). It is a misconception, which appears to be based on the work by Waterland and Jirtle [8], who had found that feeding pregnant mice with folic acid alters the phenotype (fur color and body weight) of agouti viable yellow *A*^*vy*^/*a* offspring, apparently via increased CpG methylation of an IAP retrotransposon in the *agouti* gene (*A*^*vy*^ allele) and downregulation of *agouti* transcription. These results were eagerly taken up by the epigenetics community as they appeared to provide a mechanism for the Barker hypothesis on intrauterine programming of adult disease [9]. However, the findings by Waterland and Jirtle should not be generalized and uncritically applied to humans. Of note, "provision of the same diet to wild-derived deer mice, which do not harbor variably methylated retrotransposons near the *agouti* gene, also resulted in altered *agouti*-controlled coat color …. Thus, it is possible that the increase in DNA methylation at the *A*^*vy*^ IAP is a secondary effect caused by downregulation of *agouti* transcription after methyl donor supplementation" [10].

Apart from inhibitors of chromatin modifying enzymes such as 5-azacyitidine or valproic acid ("epidrugs"), which do not normally occur in our environment, chromatin modifications are refractory to the direct influence of environmental factors [10]. What shapes the genome-wide patterns of chromatin modifications are the DNA sequence and transcription factors. Interindividual variation in DNA methylation is correlated with genetic variation [11; and references therein]. Differences in DNA methylation and histone acetylation patterns between different cell types are mainly due to differences in transcription [12, 13]. Transcriptional regulation is based on *cis*-acting DNA sequence elements (promoters, enhancers, etc.) and *trans*-acting DNA-binding factors (transcription factors, TFs); feedback loops confer stability [14]. Environmental factors affect gene expression through signaling cascades, which activate or repress TFs. Pioneer TFs "open" or "close" chromatin and thus enable or prevent other TFs to regulate gene expression. Chromatin modifying enzymes cannot read DNA sequence, but are recruited by TFs. "Although chromatin regulators are critical partners for TFs, they play a secondary role in the definition of cell fates. Rather, a primary function of chromatin during development is to reinforce or stabilize these lineages and cell fates" [15]. Thus, changes in chromatin modifications reflect rather than cause changes in gene expression. Exceptions to this rule are rare, naturally occurring epimutations (see below), experimentally induced changes in chromatin modifications ("epigenome editing") and "epidrugs", which can affect the local kinetics of gene expression. Chromatin modifications are rarely transmitted through the germline (and if so, they are hardly adaptive), but are established anew in each generation. The presence of the same chromatin modifications in parent and offspring mimics transgenerational inheritance, but in fact reflects the inheritance of the same genes and the same environment [16].

## Methodological shortcomings

The two biggest confounders of epigenetic studies are genetic variation and cell mixture distribution. Unfortunately, not all researchers are aware of these confounders. Methylation differences between cases and controls, for example, may not only be related to the phenotypic difference but also due to non-random distribution of alleles at methylation quantitative trait loci (mQTLs). The smaller the sample sizes are, the bigger this problem is. Another possibility is that the trait of interest has a genetic component and that this component also affects DNA methylation. In this case, DNA methylation may be a mediator of the genetic variant or just be an innocent bystander.

In addition to the genuine influence of the genome on DNA methylation, there are also artefactual genomic effects, at least when Illumina methylation arrays are used. These arrays are very popular, because they offer a cost-effective way of interrogating a large number of CpGs across the genome. Genetic artefacts are a general problem associated with probe-based methylation assays [17]. They result from single nucleotide polymorphisms (SNPs) around the target sites of the Illumina probes, because certain alleles of such SNPs can lead to false methylation calls. Although there are exclusion lists of unreliable probes, "current strategies to account for genetic artifacts are lagging" [17].

The second major confounder of epigenetic studies is cell mixture distribution. Since each cell type has a characteristic pattern of DNA methylation, the finding of methylation differences in complex tissues obtained from cases and controls or from the same group of probands before and after some intervention may be due to a true change in the methylation of a certain cell type or—probably more often—due to a difference in the cellular composition of the tissue. In the latter case, there is no change of DNA methylation in a cell. Typically, DNA methylation differences reflecting a different cellular composition of a tissue are small. They cannot play a causal role in the disease process, but might be used as a biomarker. There have been attempts to correct for blood cell mixture distribution (see, for example, [18]), but these methods account only for the major cell types. Better methods are currently being developed. If possible, pure cell populations should be used, but it is often very difficult to obtain such cells. My group, for example, has found that purified sperm samples of infertile men are often contaminated with varying amounts of somatic cells, which can lead to false results [19]. In this study we also found that genetic variation can confound this analysis [19].

## Clinical epigenetics

Despite the problems discussed above, there is a lot of solid evidence that epigenetic defects contribute to human disease. In 1986, Jeggo and Holiday coined the term "epimutation" [20]. In 2006, I suggested to distinguish between primary and secondary epimutations [21]. I defined a primary epimutation as an aberrant chromatin state that has occurred without any DNA sequence change, possibly as a defect in the establishment or maintenance of a particular chromatin state. Primary epimutations are rare, typically occur at a single locus and are associated with aberrant gene expression. They have been identified as one cause for imprinting defects in patients with Prader-Willi syndrome, Angelman syndrome or Beckwith–Wiedemann syndrome (see, for example, [22]). With increasing age, defects in the maintenance of DNA methylation patterns appear to occur at multiple loci in a small fraction of cells of all tissues. These defects probably provide the basis for the "epigenetic clock" [23], although the causal relationship between the DNA methylation changes, age and disease remains unclear.

Secondary epimutations result from genetic mutations. In this case, the epimutation is not the cause of the disease, but part of the mechanism by which a genetic mutation causes disease. Such a mutation can act in *cis* or in *trans*. In rare cases, for example, a disease gene is silenced and methylated as a consequence of transcriptional read through from a mutated adjacent gene. Examples include the *HBA2*, *MSH2* and *MMACHC* loci [24–26]. It should be noted that in some of these cases, methylation of the disease gene promoter was initially thought to be a hereditary primary epimutation, until the genetic defect in the adjacent gene was discovered. In other rare cases, a genetic syndrome is caused by a mutation of a gene that encodes a chromatin modifying enzyme. Such a mutation leads to aberrant chromatin states at many loci and a complex clinical phenotype. Patients with Kleefstra syndrome, for example, have a mutation in the *EHMT1* gene, which encodes the euchromatic histone lysine methyltransferase 1 [27].

Chromatin modifications cannot only be affected by a mutation in genes encoding a chromatin modifying enzyme, but also by a mutation in genes encoding other chromatin or transcriptional regulators. The chromatin changes can include changes in DNA methylation, even if no DNA modifying enzyme is affected, which substantiates the notion that changes in DNA methylation follow rather than precede other changes. Unique genomic DNA methylation patterns (called "episignatures") have been observed in a number of rare congenital diseases including the above mentioned Kleefstra syndrome and can be used for diagnostic testing [28]. Genetic mutations affecting the chromatin directly or indirectly are very frequent in cancer. As a consequence, the chromatin of tumor cells is very different from that of normal cells and can be used for stratifying tumors.

## Conclusions

While it is well established that primary and secondary epimutations contribute to human disease, there is little evidence for a direct effect of environmental factors on chromatin modifications and for transgenerational epigenetic inheritance. The epigenome is mainly shaped by the DNA sequence and by transcription. The environment affects gene expression mainly through signaling cascades and the activation or repression of transcription factors, which recruit chromatin regulators. Unfortunately, genetic variation, genetic artefacts and cell mixture distributions have not always been rigorously excluded in epigenetic studies. Mis- and overinterpretation of epigenetic data foster genetic denialism ("We can control our genes") and epigenetic determinism ("You are what your parents ate"). These erroneous beliefs can be overcome by using precise definitions, by raising the awareness about methodological pitfalls and by returning to the basic facts in molecular and cellular biology.

## Data Availability

Not applicable.

## Abbreviations

*A*: *agouti* Wildtype allele
*A*^*vy*^: *agouti* Viable yellow allele
CpG: Cytosine-guanine dinucleotide
DNA: Deoxyribonucleic acid
*EHMT1*: *Euchromatic Histone Lysine Methyltransferase 1*
*HBA2*: *Hemoglobin Subunit Alpha 2*
IAP: Intracisternal A-particle
RNA: Ribonucleic acid
miRNA: MicroRNA
*MMACHC*: *Metabolism of Cobalamin Associated C*
mQTL: Methylation quantitative trait locus
*MSH2*: *MutS Homolog 2*
SNP: Single nucleotide polymorphism
TF: Transcription factor
